# Supplementary material for: Community knowledge, attitudes, and practices toward cutaneous leishmaniasis in central Ethiopia
Source: PLoS Negl Trop Dis. 2026 Jan 5;20(1):e0013838. doi: 10.1371/journal.pntd.0013838 (PMC12844503; doi:10.1371/journal.pntd.0013838)
Supplement: S1 File — (DOCX) [file pntd.0013838.s001.docx]

An English Version of Questionnaire for the Study, 2025

Questionnaire on Cutaneous Leishmaniasis Study in Kambata Zone, Central Ethiopia

Title: Knowledge, attitude and practice of community toward cutaneous leishmaniasis Questionnaire Identification Information

Participant ID: _____________________________________________________

Name of Data Collector: _____________________________________________

Date of Data Collection: DD/MM/YY __________________________________

Time Data Collection Started: HH: MM__________________________AM/PM

Time Data Collection Ended: HH: MM__________________________AM/PM

Signature of Data Collector: __________________________________________

| **Serial number** | **Domains of the Questions** | **Categories, Codes and responses** |
| --- | --- | --- |
| **Section 1: Socio-Demographic Characteristics** | | |
| 1 | What is your sex? | 1. Male  2. Female |
| 2 | What is your age? | _________in years |
| 3 | What is your living setting? | 1. Rural  2. Urban |
| 4 | What is your occupation? | 1. Housewife  2. Farmer  3. Merchant  4. Student  5. Other (specify): |
| 5 | What is your educational status? | 1. Had formal education 2. Had no formal education |
| 6 | What is your religion? | 1. Protestant  2. Orthodox  3. Muslim  4. Catholic |
| 7 | What is your monthly household income? estimate | 1. Low  2. Medium  3. Moderate |
| 8 | Do you have a family history of cutaneous leishmaniasis? | 1. Yes  2. No |
| 9 | Have you used any medication in the past? | 1. Yes  2. No |
| **Section 2: Environmental and Housing Characteristics** | | |
| 10 | Do you have a small garden around your house? | 1. Yes  2. No |
| 11 | Do you have any domestic animals? | 1. Yes  2. No |
| 12 | Have you noticed any population movement in your area? | 1.Yes  2.No |
| 13 | Do you have a latrine in your household? | 1. Yes  2. No |
| 14 | Do you practice open defecation? | 1. Yes  2. No |
| 15 | What is the main material used for the walls of your house? | 1. Mud  2. Stone |
| 16 | Do you find any cracks in the walls of your house? confirm | 1. Yes  2. No |
| 17 | Do you use bed nets? | 1. Yes  2. No |
