# Supplementary material for: Community knowledge, attitudes, and practices toward cutaneous leishmaniasis in central Ethiopia
Source: PLoS Negl Trop Dis. 2026 Jan 5;20(1):e0013838. doi: 10.1371/journal.pntd.0013838 (PMC12844503; doi:10.1371/journal.pntd.0013838)
Supplement: Attachment — Submitted filename: 2025-10 Reviewer Report.docx [file pntd.0013838.s002.docx]

The manuscript reports data on KAP toward CL in an endemic area of Ethiopia. These data are relevant for the development and implementation of a control program in the focus. However, the manuscript cannot be published as submitted. I think that the aspects listed below should be taken into consideration in the revision for final acceptance.

- The language should be improved. For example, Line 59, the authors wrote elephantiasis talking about leishmaniasis. In addition, line 60, they have *Leishmania* that is neither italicized nor in bold. This is true for many genus or species names throughout the text that are neither italicized nor in bold. Line 70, they have leishmaniasis with upper case L.
- In the methods, the authors mentioned using 2007 Census data (Line 116). These data are 17 years old. I am not sure that the sample used in this study represents the current population of the study area. The authors must address this for the MS to be relevant.
- Sampling techniques, the authors should define for non-Ethiopians words such as Angecha and Kebele. In the text, they also use village.
- Results: Line 200, they mention the religion of the participants. However, the discussion does not	take into account the religious beliefs of respondents. A few data such as house wall material, floor and roofing material in Table 2 were not discussed at all. One would like to see a discussion of the relevance of such data in the epidemiology of CL in the focus
