# Supplementary material for: Community knowledge, attitudes, and practices toward cutaneous leishmaniasis in central Ethiopia
Source: PLoS Negl Trop Dis. 2026 Jan 5;20(1):e0013838. doi: 10.1371/journal.pntd.0013838 (PMC12844503; doi:10.1371/journal.pntd.0013838)
Supplement: Attachment — Submitted filename: Response to Reviewers (2).docx [file pntd.0013838.s004.docx]

**Response to Reviewers**

Dears, we extend our gratitude to the respected editor and reviewers for their meticulous assessment of our manuscript and the valuable feedback provided. In response to the constructive comments received, we have diligently revised the manuscript to enhance its clarity, rigor, and comprehensiveness. In the following section, we present a comprehensive point-by-point rebuttal detailing the specific actions taken to address each comment. The insightful comments from the reviewers have been instrumental in refining the quality and adherence to the journal's guidelines of our work. We are confident that the revised version reflects a significant improvement and aligns with the expectations of both the reviewers and the editorial board. Through a meticulous integration of revisions and clarifications, we have endeavored to address all concerns raised by the reviewers and indicated there in blue highlight. The input provided by the reviewers has been invaluable, and we are appreciative of the opportunity to enhance our manuscript.

**Responses to Specific Editorial Requests**

1. **CRediT Author Contribution**

- we have completed the CRediT author contribution statements for all authors, including Tagesech Yohannes. The contributions have been accurately updated in the “Add/Edit/Remove Authors” section in accordance with the journal’s guidelines and we have added Daniel Geleta (line 3).

2. **Figure Files**

- All main figures have been uploaded as individual high-resolution **.tif** files that meet the journal’s formatting specifications.

3. **Copyright Permissions**
   We have reviewed all figures to verify copyright status. For **Figure 1**, we confirm the use of an open-access Natural Earth map layer. The license information and source attribution have been added to the figure legend.

**4. Data Availability Statement**
 The Data Availability Statement has been revised to ensure complete transparency. Specifically, we now confirm that: All relevant data are within the manuscript and its supporting information files and can be accessed from author on reasonable request(line 460).

Any remaining data not included in the main text will be uploaded as Supporting Information or deposited in a recommended open repository, with links provided.

**5. Financial Disclosure**
We have updated the Financial Disclosure statement as follows: If not funded: “The authors received no specific funding for this work.” (Line 462)

**Responses to Reviewer #1 and Reviewer #2**

**Introduction**

The introduction has been revised to improve coherence and flow. We strengthened the global-to-local narrative by integrating updated WHO references and region-specific epidemiological context relevant to Ethiopia.

**Methods**

- We clarified the rationale for selecting the study period (line 122), highlighting seasonal vector activity and ecological factors influencing transmission.

**Results**

- We added explanatory text regarding participants’ familiarity with the terms “*Leishmania*” and local disease names.
- **Figure 1** has been improved with clearer regional boundaries, labels, and source details.
- Categories under “Other” in **Table 2** have been clarified to ensure interpretability (line 230).

**Statistical Considerations**

We acknowledge the limitation associated with reliance on the 2007 Census data. A clear statement addressing the potential impact on population representativeness has been added to the revised manuscript.

**Editorial and Language Revisions**

- All incorrect references to “elephantiasis” have been replaced with “cutaneous leishmaniasis.”
- All genus and species names have been italicized following standard taxonomic conventions.
- Local administrative terms such as “Angecha,” “Kebele,” and “village” have been defined for the benefit of international readers (line 131-2).
